# GAGA Regulates Border Cell Migration in *Drosophila*

**DOI:** 10.3390/ijms21207468

**Published:** 2020-10-10

**Authors:** Anna A. Ogienko, Lyubov A. Yarinich, Elena V. Fedorova, Natalya V. Dorogova, Sergey I. Bayborodin, Elina M. Baricheva, Alexey V. Pindyurin

**Affiliations:** 1 Department of the Regulation of Genetic Processes, Institute of Molecular and Cellular Biology of the Siberian Branch of the Russian Academy of Sciences, 630090 Novosibirsk, Russia; l.yarinich@mcb.nsc.ru; 2 Faculty of Natural Sciences, Novosibirsk State University, 630090 Novosibirsk, Russia; 3 Department of Cell Biology, Institute of Cytology and Genetics of the Siberian Branch of the Russian Academy of Sciences, 630090 Novosibirsk, Russia; fedorova@bionet.nsc.ru (E.V.F.); dorogova@bionet.nsc.ru (N.V.D.); bai@bionet.nsc.ru (S.I.B.); barich@bionet.nsc.ru (E.M.B.)

**Keywords:** *Drosophila melanogaster*, cell migration, border cells, *slbo*, *Trl*, GAGA

## Abstract

Collective cell migration is a complex process that happens during normal development of many multicellular organisms, as well as during oncological transformations. In *Drosophila* oogenesis, a small set of follicle cells originally located at the anterior tip of each egg chamber become motile and migrate as a cluster through nurse cells toward the oocyte. These specialized cells are referred to as border cells (BCs) and provide a simple and convenient model system to study collective cell migration. The process is known to be complexly regulated at different levels and the product of the *slow border cells* (*slbo*) gene, the C/EBP transcription factor, is one of the key elements in this process. However, little is known about the regulation of *slbo* expression. On the other hand, the ubiquitously expressed transcription factor GAGA, which is encoded by the *Trithorax-like* (*Trl*) gene was previously demonstrated to be important for *Drosophila* oogenesis. Here, we found that *Trl* mutations cause substantial defects in BC migration. Partially, these defects are explained by the reduced level of *slbo* expression in BCs. Additionally, a strong genetic interaction between *Trl* and *slbo* mutants, along with the presence of putative GAGA binding sites within the *slbo* promoter and enhancer, suggests the direct regulation of this gene by GAGA. This idea is supported by the reduction in the *slbo*-Gal4-driven GFP expression within BC clusters in *Trl* mutant background. However, the inability of *slbo* overexpression to compensate defects in BC migration caused by *Trl* mutations suggests that there are other GAGA target genes contributing to this process. Taken together, the results define GAGA as another important regulator of BC migration in *Drosophila* oogenesis.

## 1. Introduction

Collective cell migration was found in many different organisms during embryonic development and wound healing, as well as in some metastatic cancers. In *Drosophila* oogenesis, a small set of follicle cells (FCs) originally located at the anterior tip of each egg chamber become motile and migrate as a cluster through nurse cells toward the oocyte. These specialized cells are referred to as border cells (BCs) and provide a simple and convenient model system to study the mechanisms that control collective cell migration in vivo [1,2,3,4]. More specifically, BCs arise within an epithelium consisting of more than a thousand FCs that encircle a cluster of 16 germline cells to form an egg chamber [5]. At early stage of oogenesis, two specialized FCs, referred to as polar cells, differentiate both at the anterior and posterior tips of the egg chamber [6,7,8]. The anterior polar cells recruit several (from 4 to 8) additional neighboring FCs, and the cluster becomes motile, invades the group of nurse cells and moves toward the oocyte [9].

A number of genes are known to be necessary for the conversion of BCs from stationary epithelial cells into motile, invasive cells. The specification and movement of BCs are controlled by different signaling pathways, ecdysone steroid hormone, receptor tyrosine kinases as well as by multiple transcription factors [10]. The polar cells secrete cytokines, which bind to a transmembrane receptor of neighboring FCs to trigger activation of Janus Kinase (JAK) [11,12,13,14,15]. JAK phosphorylates Signal Transduction and Activator of Transcription (STAT), which then translocates to the nucleus, where it activates transcription of a number of target genes [16,17,18]. Among those is the *slow border cells* (*slbo*) gene, which encodes a C/EBP transcription factor (hereafter Slbo), that determines BC fate [9]. Slbo is responsible for regulation of expression of numerous factors implicated in the cell migration process, such as cytoskeletal regulators and adhesion molecules. Eventually, sufficient amount of the Slbo protein must be present in BCs for their movement [19,20].

Only a few factors are known that regulate the *slbo* gene expression at the transcriptional level. Namely, STAT, Traffic jam and Notch via Su(H) were found to be necessary for activation of the *slbo* gene transcription [13,21,22,23,24], whereas Apontic, Bunched, Cut, Hindsight and Slbo itself were demonstrated to downregulate *slbo* activity [21,23,25,26,27,28]. However, studies on the interactions between these transcription factors and their putative binding sites within *slbo* regulatory sequences are limited and were performed solely by using in vitro assays [21,26]. To simplify the analysis of BC behavior, the *slbo*-Gal4 driver construct was previously constructed, in which Gal4 expression was placed under the control of a minimal promoter element and a 2.6-kb *slbo* enhancer [29]. This construct was integrated at different genomic sites and currently a number of *slbo*-Gal4 driver lines are available (e.g., chic6458, Novo11, Novo16 and Novo22) that express Gal4 in a pattern indistinguishable from that of the endogenous Slbo [29,30].

Previously, we reported that the ubiquitously expressed product of the *Trithorax-like* (*Trl*) gene, the transcription factor GAGA (also known as GAGA factor or GAF), is required for normal *Drosophila* oogenesis [31,32,33]. GAGA is well known for regulation of transcriptional activity of many genes through local modifications of chromatin structure at their regulatory elements [34,35,36,37,38]. In developing egg chambers, GAGA is present in the nucleus of all cells and the decrease in the protein level results in defects in actin cytoskeleton organization, the disruption of oogenesis and reduced female fertility [31,32,33,39].

Here, we found that *Trl* mutations cause substantial defects in BC migration, which can be partially explained by the direct regulation of *slbo* transcriptional activity by GAGA. The results obtained also suggest that there are other GAGA target genes contributing to the cell migration process. Altogether, GAGA can be defined as an important regulator of BC migration in *Drosophila* oogenesis.

## 2. Results

### 2.1. Molecular Characterization of Chromosomes Carrying Trl^362^ and Trl^3609^ Mutations

First, we verified fly stocks with *Trl* mutations chosen for the study, *Trl^362^*, *Trl^3609^*, *Trl^13C^* and *Trl^R85^*, by genotyping PCR with allele-specific primers (Table S1). In addition, since according to our previous experience, transgenic *Drosophila* lines frequently bear extra uncharacterized transposon constructs, sometimes even within genes relevant to the studied process [30], we checked the *Trl* mutant lines for the presence of additional *P* element sequences by quantitative PCR. Indeed, this analysis demonstrated that chromosomes carrying *Trl^362^* and *Trl^3609^* mutations carry extra *P* element transgenes, one in each case (Figure S1A). The subsequent inverse-PCR mapping of *P* element insertion sites in *Trl^362^* and *Trl^3609^* lines revealed previously unknown additional transgenic constructs located within the *alan shepard* (*shep*) and *couch potato* (*cpo*) genes, respectively (Figure S1B). The transposon inserted in the intron/promoter of the *shep* gene (3L: 5,248,444–5,248,451; here and afterwards, coordinates are from Release 6 of the *Drosophila melanogaster* genome assembly [40]) consists almost exclusively of *P* element end sequences and has a total length of 913 bp. The transgene inserted in the intron/promoter of the *cpo* gene (3R: 17,944,070–17,944,077) is much longer (about 9 kb) and its internal composition (DNA sequence between *P* element ends) was not investigated. The presence of these novel transgenes in *Trl^362^* and *Trl^3609^* lines was confirmed by PCR with primers specific to the *P* element ends and the sequences flanking the insertion sites (Figure S1B, Table S1).

### 2.2. Decrease in Trl Expression Delays BC Migration

**Figure 1:**
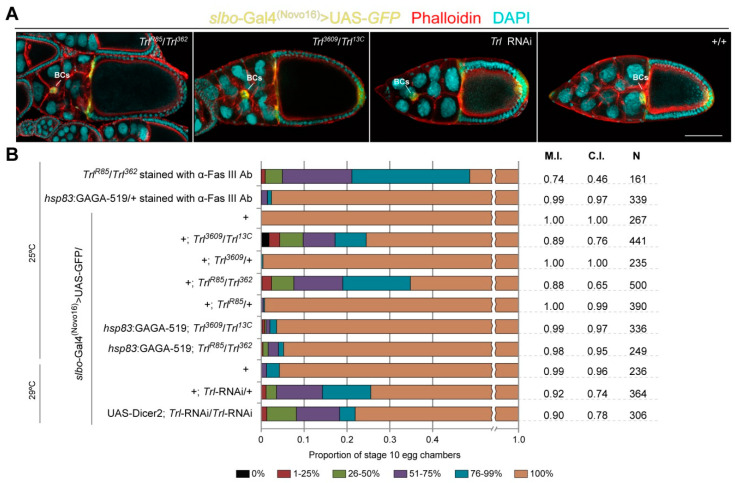
Border cell (BC) migration is delayed in *Trithorax-like* (*Trl*) mutant egg chambers. (**A**) Stage 10 egg chambers from *Trl* mutant and *Trl^+/+^* flies labelled with *slbo*-Gal4 > UAS-*GFP* (yellow), Phalloidin (red) and DAPI (cyan). Note that in the shown examples of *Trl* mutant egg chambers, BCs had not reached the nurse cell–oocyte boundary. Scale bar is 100 µm. (**B**) Quantification of the BC migration phenotypes in stage 10 egg chambers of the indicated genotypes. The combination of the *slbo*-Gal4^(Novo16)^ driver [30] with the UAS-*GFP* reporter construct or immunostaining with anti-Fasciclin III antibody (α-Fas III Ab) was used to mark BCs. UAS-Dicer2 was used to increase efficiency of RNAi. “+” indicates wild-type second or third chromosome(s) depending on the genotype. For quantitation, the nurse cell region of egg chambers was divided into 6 groups according to the percentage of the total distance travelled by BCs: 0% (black), 1–25% (dark red), 26–50% (light green), 51–75% (violet), 76–99% (blue) and 100% (brown) [30]. M.I., C.I. and N denote the migration index, the completion index and the number of egg chambers examined, respectively.

We examined whether the GAGA protein is important for BC migration. For that, we studied this process in hypomorphic *Trl* mutants, *Trl^R85^*/*Trl^362^* and *Trl^3609^*/*Trl^13C^*. A significant delay in BC migration was observed in both mutant combinations compared to the control (Figure 1A). Particularly, the migration and completion indexes, parameters that characterize the distance covered by BCs by the end of stage 10 [41], were much lower in *Trl^R85^*/*Trl^362^* and *Trl^3609^*/*Trl^13C^* mutants than in *Trl^+^*/*Trl^+^* flies (Figure 1B). These defects were almost completely rescued by ubiquitous overexpression of GAGA by the means of the *hsp83*: GAGA-519 transgene [42] in the *Trl^R85^*/*Trl^362^* and *Trl^3609^*/*Trl^13C^* mutant backgrounds (Figure 1B) pointing to the necessity of this transcription factor for BC migration. Notably, the overexpression of GAGA per se had very little effect on migration of BCs (Figure 1B). To assess the input of BC-specific expression of the *Trl* gene in the observed phenomenon, we induced RNA interference (RNAi) of this gene using the *slbo*-Gal4 driver, which is exclusively active in BCs, posterior and centripetal FCs [29,30]. This resulted in BC migration defects similar to those observed in *Trl^R85^*/*Trl^362^* and *Trl^3609^*/*Trl^13C^* mutants (Figure 1B). Thus, we concluded that proper migration of BCs depends on the level of the GAGA protein.

### 2.3. GAGA Regulates Transcriptional Activity of the slbo Gene during Migration of BCs

**Figure 2:**
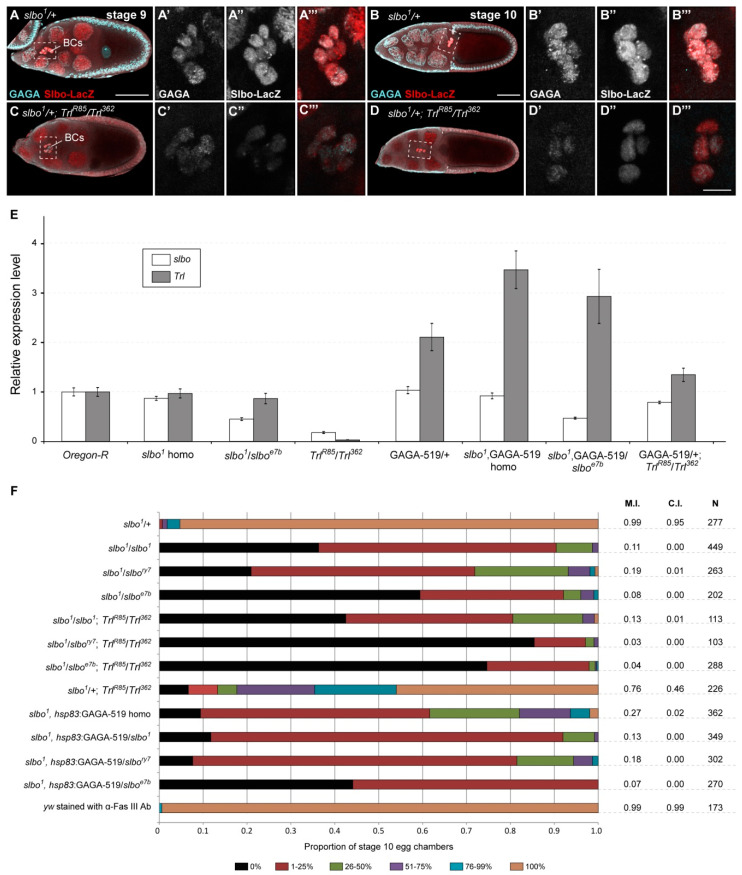
Downregulation of GAGA decreases the activity of the *slow border cells* (*slbo*) gene in migrating BCs. (**A**–**D**) Colocalization of β-galactosidase driven by the *slbo-LacZ* reporter (*slbo^1^* enhancer trap mutation) and the GAGA protein in BCs. Anti-GAGA (cyan) and anti-β-galactosidase (red) immunostaining of *slbo^1^*/+ (**A**,**B**) and *slbo^1^*/+; *Trl^R85^*/*Trl^362^* (**C**,**D**) egg chambers at stages 9 (**A**,**C**) and 10 (**B**,**D**). Note the reduction in GAGA and β-galactosidase signals in BCs of *Trl* mutants. The regions of BCs are marked by dotted frames and are enlarged in **A′**–**A‴**, **B′**–**B‴**, **C′**–**C‴** and **D′**–**D‴**, in which single cyan (**A′**, **B′**, **C′** and **D′**) and red (**A″**, **B″**, **C″** and **D″**) channel images, as well as merge images (**A‴**, **B‴**, **C‴** and **D‴**), are shown. All egg chambers were imaged at the same settings. Scale bar is 50 µm and 10 µm for A, C, B, D and the enlargements, respectively. (**E**) Comparison of the *slbo* and *Trl* mRNA levels (determined by RT-qPCR) in ovaries of the indicated genotypes. Error bars represent standard error of the mean. GAGA-519 denotes *hsp83*:GAGA-519. (**F**) Downregulation of GAGA enhances BC migration defects observed in *slbo* mutants. “homo” indicates homozygous state. Quantification of the BC migration phenotypes in stage 10 egg chambers of the indicated genotypes. X-gal staining was used to visualize BCs in all samples except the *yw* control, which was immunostained with anti-Fasciclin III antibody (α-Fas III Ab). For quantitation, the nurse cell region of egg chambers was divided into 6 groups according to the percentage of the total distance travelled by BCs: 0% (black), 1–25% (dark red), 26–50% (light green), 51–75% (violet), 76–99% (blue) and 100% (brown) [30]. M.I., C.I. and N denote the migration index, the completion index and the number of egg chambers examined, respectively.

Next, we wondered whether the GAGA protein is expressed in BCs along their migration to the nurse cell–oocyte boundary. To assess that, we used the *slbo^1^* allele caused by the insertion of *LacZ* reporter gene within the promoter region of the *slbo* gene [9]; this mutation is also known as *slbo-LacZ*. It was previously demonstrated that the β-galactosidase expression pattern driven by *slbo^1^* is matching that of the endogenous Slbo protein. Importantly, heterozygous *slbo^1^* egg chambers develop normally and morphologically are indistinguishable from the wild-type counterparts [9]. Immunostaining of *slbo^1^*/+ stage 9 and stage 10 egg chambers with anti-β-galactosidase and anti-GAGA antibodies revealed that these proteins colocalize in the BC nuclei along the entire process of cell migration; while GAGA was also detected in all FCs (Figure 2A,B). Furthermore, the reduction in the GAGA protein level in *slbo^1^*/+; *Trl^R85^*/*Trl^362^* mutants led to a pronounced decrease in β-galactosidase staining at both analyzed stages of egg chamber development, suggesting the regulation of the *slbo* gene activity by GAGA (Figure 2C,D).

To check this hypothesis, we used RT-qPCR to measure the abundance of *slbo* and *Trl* transcripts in ovaries of different genotypes (Figure 2E). Indeed, a strong reduction in *slbo* transcripts (down to ~20%) was detected in *Trl^R85^*/*Trl^362^* ovaries. At the same time, *slbo* transcript levels were much less affected in hypomorphic *slbo^1^*/*slbo^1^* and *slbo^1^*/*slbo^e7b^* mutants. In addition, overexpression of GAGA had no obvious effect on the *slbo* gene transcription (Figure 2E). Taken together, the *slbo* gene expression in migrating BCs appears to be regulated by the GAGA protein.

### 2.4. Genetic Interaction between Trl and slbo Genes

To further study the possible regulation of the *slbo* gene expression by GAGA, we employed the genetic approach. Specifically, we compared BC migration defects observed in hypomorphic *slbo* mutants with those demonstrated by hypomorphic *slbo* and *Trl* double mutants. Analysis of stage 10 egg chambers from *slbo^1^*/*slbo^1^* and *slbo^1^*/*slbo^1^*; *Trl^R85^*/*Trl^362^* flies showed that BC migration was completely blocked in 36% and 42% of cases, respectively (Figure 2F). Similarly, the frequency of such strong defects observed in *slbo^1^*/*slbo^ry7^* and *slbo^1^*/*slbo^e7b^* egg chambers increased, respectively, from 21% to 85% and from 59% to 75% in *Trl^R85^*/*Trl^362^* mutant background (Figure 2F). On the contrary, the addition of one copy of the *hsp83*: GAGA-519 transgene slightly rescued the complete blockage of BC migration observed in *slbo* mutants; the defect was observed in 12%, 8% and 44% of *slbo^1^*, *hsp83*:GAGA-519/*slbo^1^*, *slbo^1^*, *hsp83*:GAGA-519/*slbo^ry7^* and *slbo^1^*, *hsp83*:GAGA-519/*slbo^e7b^* egg chambers, respectively (Figure 2F). Overall, the results indicate that BC migration defects observed in *slbo* mutants are severely enhanced by mutations in the *Trl* gene, whereas GAGA overexpression slightly diminishes these defects.

### 2.5. Transcriptional Activity of the slbo-Gal4 Drivers Depends on the GAGA Protein Level

**Figure 3:**
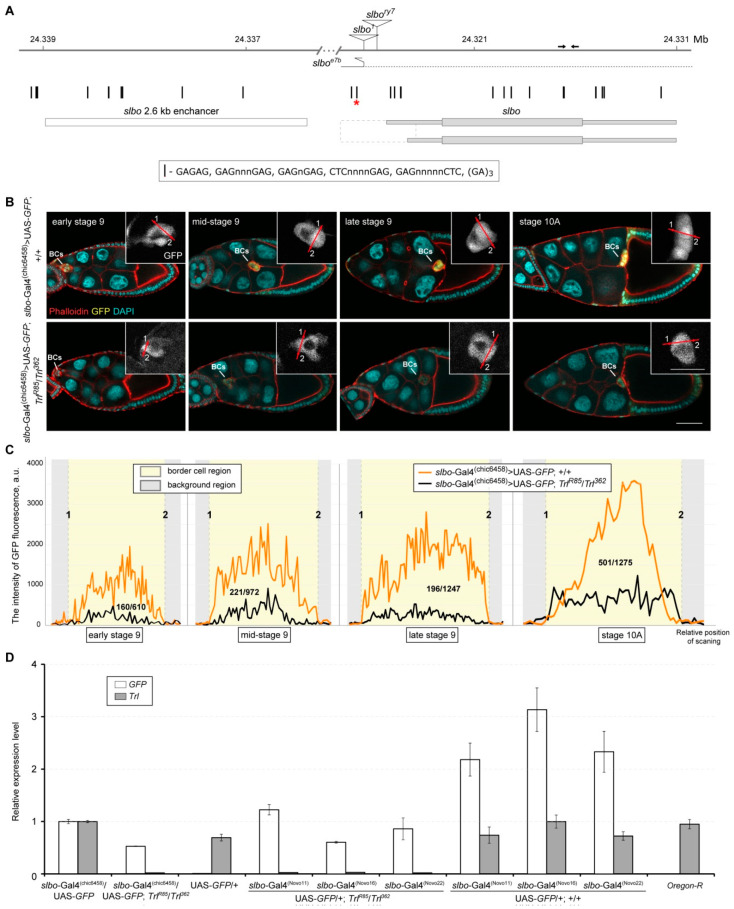
*slbo*-Gal4-driven GFP expression is dependent on GAGA. (**A**) A schematic map of the *slbo* locus on chromosome 2R. The gene is shown on the plus strand for convenience; its coding sequence and UTRs are represented by wide and narrow grey bars, respectively. The 2.6-kb enhancer element is depicted as white box. Predicted GAGA binding sites are shown by vertical sticks. Red asterisk marks the site, for which GAGA binding was previously demonstrated in in vitro experiments [43]. Putative promoter region is depicted by dashed rectangle. Localization of *P* element insertions causing the *slbo^1^* and *slbo^ry7^* mutations are shown by triangles (not drawn to scale). The position of the *slbo^e7b^* deletion (in which a fragment of *P* element is retained) is indicated by the dotted horizontal line. Arrows depict the position of primer pairs used for measurements of mRNA abundance with RT-qPCR. (**B**) Representative confocal images of egg chambers at four consecutive stages of development (early, mid and late stage 9, and stage 10A) from *slbo*-Gal4^(chic6458)^ > UAS-*GFP* and *slbo*-Gal4^(chic6458)^ > UAS-*GFP*; *Trl^362^*/*Trl^R85^* flies. Mutations in the *Trl* gene lead to significant decrease in GFP signal in BCs at all analyzed stages. All egg chambers were imaged at the same settings, scale bar is 50 µm. Insets show enlarged overexposed fragments to highlight BC cluster boundaries, scale bar is 25 µm; lines with dots 1 and 2 indicate sections used for the quantification of the GFP signal intensity. (**C**) The GFP fluorescence intensity profiles obtained for sections through BCs shown in (B). For each developmental stage, the ratio of average intensity of the GFP signal in the *Trl* mutant to that in the control is shown as a fractional number. (**D**) Effects of GAGA downregulation on *slbo*-Gal4-driven *GFP* expression (measured by RT-qPCR) in ovaries of the indicated genotypes. Error bars represent standard error of the mean. Note that in *Trl* mutant background, *GFP* expression was substantially decreased for all tested *slbo*-Gal4 drivers (chic6458, Novo11, Novo16 and Novo22).

As GAGA is a sequence-specific transcription factor, the most straightforward mechanism of its involvement in the regulation of the *slbo* gene expression would be the direct protein binding to its recognition site(s) within the target gene regulatory elements followed by local chromatin remodeling [35]. Therefore, we searched for the potential GAGA binding sites (the GAGAG, GAGnnnGAG, GAGnGAG, CTCnnnGAG, GAGnnnnnCTC and (GA)_3_ motifs [43]) within the *slbo* gene locus. Several GAGA motifs were found within the *slbo* promoter region as well as within the previously described 2.6-kb enhancer element (Figure 3A), which is present in *slbo*-Gal4 drivers chic6458, Novo11, Novo16 and Novo22 [29,30]. Due to availability of the drivers, we decided to check whether their functioning depends on the amount of the GAGA protein. To this end, we first compared the intensities of the *slbo*-Gal4-driven GFP signals within BC clusters of stages 9 and 10 egg chambers in the wild-type and *Trl^R85^*/*Trl^362^* mutant backgrounds (Figure 3B,C). This analysis demonstrated that, on average, the GFP fluorescence intensity was about 4.4 times lower in *Trl* mutants than in the control. In addition, results of RT-qPCR revealed that the *GFP* expression driven by different *slbo*-Gal4 drivers was reduced from 1.8- to 5.2-fold in *Trl* mutant background (Figure 3D). Thus, we concluded that GAGA appears to regulate the transcriptional activity of the *slbo*-Gal4 drivers containing the 2.6-kb enhancer element.

### 2.6. Increase in the slbo Expression Level in Trl Mutants Enhances BC Migration Defects

**Figure 4:**
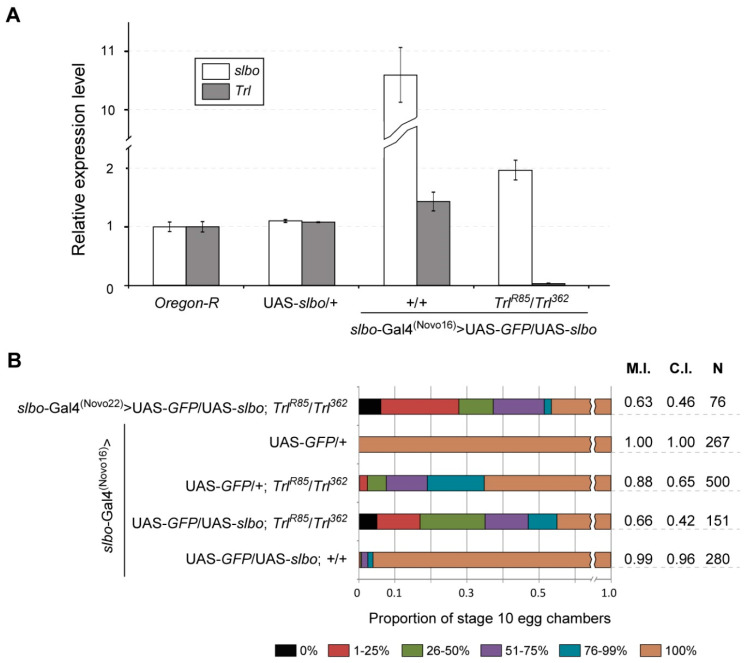
Overexpression of Slbo does not rescue impaired BC migration in *Trl* mutants. (**A**) Comparison of the *slbo* and *Trl* mRNA levels (determined by RT-qPCR) in ovaries of the indicated genotypes. The data for the *Oregon-R* control are the same as in Figure 2E. Error bars represent standard error of the mean. (**B**) Quantification of the BC migration phenotypes in stage 10 egg chambers of the indicated genotypes. The combination of the *slbo*-Gal4^(Novo16)^ or *slbo*-Gal4^(Novo22)^ driver [30] with the UAS-*GFP* reporter construct was used to mark BCs. For quantitation, the nurse cell region of egg chambers was divided into 6 groups according to the percentage of the total distance travelled by BCs: 0% (black), 1–25% (dark red), 26–50% (light green), 51–75% (violet), 76–99% (blue) and 100% (brown) [30]. M.I., C.I. and N denote the migration index, the completion index and the number of egg chambers examined, respectively.

Since *slbo* seems to be a target gene for GAGA, we wondered whether *slbo* overexpression can rescue BC migration defects observed in *Trl* mutants. To check this, we overexpressed exogenous copy of the *slbo* coding sequence (UAS-*slbo*) in BCs using the *slbo*-Gal4^(Novo16)^ driver. In a wild-type background, this led to about 10.5-fold increase in *slbo* mRNA level and resulted only in a minimal disruption of the collective cell migration process (Figure 4), which is principally consistent with earlier observations [25]. In *Trl^362^*/*Trl^R85^* mutants, *slbo*-Gal4^(Novo16)^-driven *slbo* overexpression increased the expression level of the gene only about 2.0-fold (Figure 4A). Surprisingly, this was accompanied by stronger defects of BC migration than in *Trl^362^*/*Trl^R85^* mutants alone (Figure 4B). The same effect was observed when *slbo*-Gal4^(Novo22)^ driver was used (Figure 4B). Taken together, these results suggest that the decrease in the *slbo* expression in *Trl* mutants is not the only reason for the observed defects in BC migration. Most likely, there are some other GAGA target genes, which expression levels are crucial for the studied process.

### 2.7. Trl Expression Does Not Depend on Slbo

**Figure 5:**
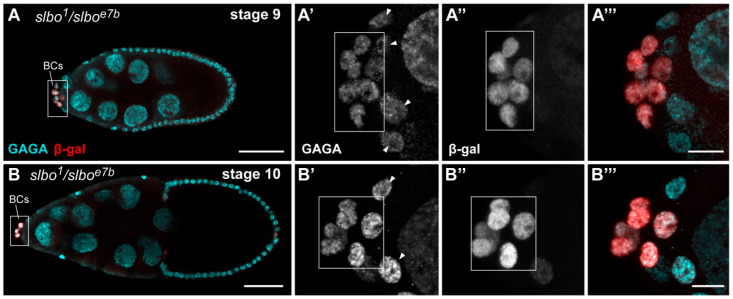
Expression of GAGA is not affected in *slbo* mutant egg chambers. Immunodetection of β-galactosidase (red) driven by the *slbo-LacZ* reporter (*slbo^1^* enhancer trap mutation) and the GAGA protein (cyan) in *slbo^1^*/*slbo^e7b^* mutant egg chambers at stages 9 (**A**) and 10 (**B**). The regions of BCs are marked by dotted frames and are enlarged in **A′**–**A‴** and **B′**–**B‴**, in which single cyan (**A′** and **B′**) and red (**A″** and **B″**) channel images as well as merge images (**A‴** and **B‴**) are shown. Scale bar is 50 µm and 10 µm for A, B, and the enlargements, respectively. As can be seen in (**A′**) and (**B′**), the amount of GAGA in *slbo* mutant BCs is not decreased compared to the adjacent follicular cells (marked with arrowheads).

Considering strong genetic interaction between *slbo* and *Trl* genes and the fact that Slbo is also a sequence-specific transcription factor [9,26], we asked whether the *Trl* gene could be a target of Slbo. To answer this question, we measured the *Trl* gene expression in *slbo* mutant ovaries by two different approaches. First, RT-qPCR measurements showed that the *Trl* expression was not substantially affected in *slbo^1^*/*slbo^1^* and *slbo^1^*/*slbo^e7b^* mutant ovaries (Figure 2E). However, this result is not very informative since the *slbo* gene is known to be active only in a minor fraction of *Drosophila* ovarian cells [29,30]. Second, we immunostained *slbo^1^*/*slbo^e7b^* egg chambers, in which *slbo* expression is decreased by 2.2-fold (Figure 2E), with anti-GAGA antibodies to estimate the amount of this protein. This assay also did not detect any obvious change in the amount of the GAGA protein in BCs and in other cell types of *slbo^1^*/*slbo^e7b^* mutants at stage 9 (Figure 5A) or at stage 10 (Figure 5B) compared to the appropriate controls (Figure 2A–D).

## 3. Discussion

Collective cell migration plays significant roles in normal development and tumorigenesis [44,45,46,47]. Therefore, thorough understanding of regulation of this process is very important. In this study, we found that along with reduced female fecundity due to egg chamber apoptosis prior to oocyte maturation [32], *Trl* mutants also demonstrate strong defects in BC migration. Particularly, different *Trl* allele combinations lead to defects in 35–49% of stage 10 egg chambers. The presence of additional *P* element insertions in the chromosomes carrying *Trl^362^* and *Trl^3609^* mutations, which were revealed in this study, most likely does not substantially influence the BC migration process due to the following two reasons. First, the rescue experiments with the *hsp83*:GAGA-519 transgene clearly show that it is the lowered amount of GAGA that is responsible for BC migration defects observed in *Trl* mutants. Second, *shep* and *cpo* affected by the additional transposon insertions have not been so far identified as BC- or ovary-specific genes. However, the additional molecular features of the *Trl^362^*- and *Trl^3609^*-bearing chromosomes should be taken into account in experiments on cells/tissues expressing these genes, such as neurons and/or glial cells in the central nervous system (CNS) [48,49,50,51].

Considering the importance of Slbo [9] as one of the main regulators of BC migration, it was interesting to assess whether Slbo and GAGA may have a functional relationship during this process. Indeed, the decrease in GAGA level in BCs results in substantial decrease in *slbo* activity, but not vice versa. At the same time, overexpression of GAGA has no effect on *slbo* mRNA level indicating that GAGA regulates *slbo* expression only positively. A strong genetic interaction between *Trl* and *slbo* mutants, along with the presence of putative GAGA binding sites within the *slbo* promoter and enhancer sequences, suggests the direct regulation of this gene by GAGA. The reduction in the *slbo*-Gal4-driven GFP expression within BC clusters in *Trl* mutant background supports this idea. It is worth noting that no putative GAGA binding sites were found within the minimal *hsp70* promoter element present in the *slbo*-Gal4 construct. The inability of *slbo* overexpression to compensate defects in BC migration caused by *Trl* mutations indicates that there are other GAGA target genes contributing to this process. Alternatively, Slbo could require GAGA and/or some other co-factor(s), which are downregulated in *Trl* mutants, to properly activate transcription of its target genes. Taken together, the results of this study define GAGA as another important regulator of BC migration in *Drosophila* oogenesis.

## 4. Materials and Methods

### 4.1. Fly Stocks

Flies were maintained on standard fly food and crossed at 25 °C except for the RNAi experiments that were performed at 29 °C according to [52]. The following lines from the Bloomington Drosophila Stock Center (BDSC; Bloomington, IN, USA; https://bdsc.indiana.edu) were used: #6458 (w*; P{*w^+mC^* = Gal4-*slbo*.2.6}1206 P{*w^+mC^* = UAS-*GFP*.S65T}*Myo31DF^T2^*) as the source of the *slbo*-Gal4-*chic^6458^* driver [29,30] (here referred to as *slbo*-Gal4^(chic6458)^); #76363 (w*; P{*w^+mC^* = Gal4-*slbo*.2.6}16, P{*y^+t7.7^ w^+mC^* = 10 × UAS-*IVS-mCD8::GFP*}attP40) as the source of the *slbo*-Gal4^(Novo16)^, UAS-*GFP* transgene combination [30]; #32186 (w*; P{*y^+t7.7^ w^+mC^* = 10 × UAS-*IVS-mCD8::GFP*}attP40) as the source of the UAS-*GFP* reporter construct; #58473 (*w^1^*; *Trl^13C^*/TM6B, *Sb^1^ Tb^1^*) as the source of the *Trl^13C^* mutation [34]; #64190 (y^1^ w*; P{*w^+mC^* = lacW}*Trl^362^*/TM3, *Sb^1^*, *Ser^1^*, *y^+^*) as the source of the *Trl^362^* mutation [31]; #10740 (P{*ry^+t7.2^* = *ry*^11^}*slbo^ry7^ cn^1^*/CyO; *ry^506^*) as the source of the *slbo^ry7^* mutation [9]; #12227 (P{*ry^+t7.2^* = PZ}*slbo^01310^ cn^1^*/CyO; *ry^506^*) as the source of the *slbo^1^* mutation, which is also known as *slbo-LacZ* [9]; #58686 (*slbo^e7b^*/CyO; *ry^506^*) as the source of the *slbo^e7b^* deletion covering the entire *slbo* coding region [26]; #41582 (*y^1^v^1^*; P{*y^+t7.7^ v^+t1.8^* = TRiP.GL00699}attP2) as the source of the RNAi construct against the *Trl* gene; #24650 (*w^1118^*; P{*w^+mC^* = UAS-Dcr-2.D}2) as the source of the UAS-Dicer2 transgene; #24482 (*y^1^* M{*vas*-int.Dm}ZH-2A w*; M{3xP3-RFP.attP’}ZH-51C) as the source of the PhiC31 integrase and the attP landing site 51C [53]; #25211 (Oregon-R-modENCODE) as the wild-type (“*Oregon-R*”) control. Fly strains with *Trl^R85^* and *Trl^EP3609^* (here referred to as *Trl^3609^*) mutations [34,54], the *hsp83*: GAGA-519 transgene [42], the *slbo*-Gal4^(Novo11)^ and *slbo*-Gal4^(Novo22)^ drivers [30] as well as *yw* (the “wild-type” control) and *yw*; *Kr^If-1^*/*CyO*; TM6, *Tb*/*Sb* stocks were taken from our laboratory collection.

### 4.2. Identification and Verification of P Element Transgene Insertion Sites

Genomic DNA was isolated from 50 flies according to the protocol described previously [55]. Determination of copy number of *P* element end sequences, mapping and verification of *P* element-based transgene insertion sites were performed according to [30] with the following modifications. Only primers specific for *P* element 5′ end and the reference *Vps36* gene were used for quantitative real-time PCR. Templates for inverse PCR were prepared using MspI (SibEnzyme, Novosibirsk, Russia) and Kzo9I (SibEnzyme) restriction enzymes. Sequences of primers used to verify *P* element insertion sites by PCR are listed in Table S1.

### 4.3. Generation of UAS-slbo Transgenic Flies

To make pUASTattB-slbo construct for ectopic expression of the Slbo protein, we first PCR-amplified the full-length *slbo* coding sequence with primers 5ʹ-AAAGAATTCCAAAATGCTGAACATGGAGTCGC-3ʹ and 5ʹ-AAATCTAGACTACAGCGAGTGTTCGTTGG-3ʹ using genomic DNA isolated from *yw* flies as a template. Next, the amplified DNA fragment was cloned into the pUASTattB plasmid vector [53] by using the unique EcoRI and XbaI sites (underlined in the primer sequences). The pUASTattB-slbo construct was verified by Sanger sequencing that revealed several synonymous nucleotide substitutions within the *slbo* coding sequence. The plasmid was injected at the concentration of 250 ng/µl into embryos of the BDSC line #24482 as described in [56].

### 4.4. Immunofluorescent Staining

Dissected ovaries were mounted on glass slides in mounting medium (50% glycerol, 0.82 mM KH_2_PO_4_, 2.6 mM NaH_2_PO_4_, 75 mM NaCl). Immunostainings were performed as described previously [57]. The primary antibodies were mouse anti-Fasciclin III (anti-Fas III; 5 µg/mL; #7G10; Developmental Studies Hybridoma Bank (DSHB), Iowa City, IA, USA), mouse anti-beta-galactosidase (anti-β-gal; 5 µg/mL; #40-1a; DSHB) and rabbit anti-GAGA (1:500; kindly provided by Prof. Vincenzo Pirrotta). The secondary antibodies were goat anti-mouse conjugated to Alexa Fluor 488 (1:500; #A-11001; Invitrogen, Carlsbad, CA, USA), goat anti-mouse Alexa Fluor 568 (1:500; #A-11031; Invitrogen) and goat anti-rabbit Alexa Fluor 488 (1:500; #A-11034; Invitrogen). TRITC-labeled phalloidin (1:100; #P1951; Sigma-Aldrich, Saint Louis, MO, USA) was used to visualize F-actin as described previously [58]. DAPI was used at 0.4 µg/mL to stain nuclei. Samples were imaged using an Axio Observer Z1 (Carl Zeiss, Oberkochen, Germany) and confocal microscope LSM 710 (Carl Zeiss). Optical sections were combined using the LSM Image Browser version 4.2 software (Carl Zeiss).

### 4.5. Quantitative Measurement of GFP Signal Intensity

The GFP signals were detected using confocal microscope LSM 710 (Carl Zeiss). Fluorescence intensity quantification was performed for individual confocal images acquired at the same settings using the ZEN 2012 software v 8.1. Experiments were performed in three biological replicates for each genotype and stage of egg chamber development.

### 4.6. Detection of BCs and Quantitative Analysis of Their Migration

BCs were identified either by the *slbo*-Gal4-driven GFP expression or by immunostaining with anti-Fas III antibodies (that reveal polar but not outer BCs [6]) or by X-gal (5-bromo-4-chloro-3-indolyl-β-D-galactopyranoside; #A1007.0001; BioChemica, AppliChem, Germany) staining of *slbo^1^* (*slbo-LacZ*) mutants. The latter procedure was performed as follows. *Drosophila* ovaries were dissected in 1×PBS (1.7 mM KH_2_PO_4_, 5.2 mM Na_2_HPO_4_, 150 mM NaCl; pH 7.4) and then fixed in 0.75% glutaraldehyde (#G5882; Merck, Darmstadt, Germany) in 100 mM sodium cacodylate buffer (pH 7.0) (#A2140; PanReac AppliChem, Chicago, IL, USA) for 20 min at room temperature. Next, the ovaries were incubated in a staining solution (10 mM sodium phosphate (pH 7.2), 3.1 mM K_4_[Fe(CN)_6_], 3.1 mM K_3_[Fe(CN)_6_], 150 mM NaCl, 1.0 mM MgCl_2_, 0.2% X-gal, 0.3% Triton X-100) for 1 h at 37 °C. The migration and completion indexes characterizing BC migration process were calculated as described previously [30].

### 4.7. Total RNA Extraction, cDNA Synthesis and Quantitative Real-Time PCR

For each genotype, three replicates of 50 ovaries from 1–2-day-old flies were dissected in 1×PBS, preserved in 100 µL of RNAlater solution (#AM7020; Thermo Fisher Scientific, Waltham, MA, USA) and stored at 4 °C. Subsequent isolation of total RNA, reverse transcription and quantitative PCR (RT-qPCR) were carried out as reported previously [30] with primer pairs specific for *A. vinelandii GFP* coding sequence and *Drosophila slbo*, *Trl*, *RpL32* and *Rap2l* genes (for primer sequences, see Table 1). The latter two genes were used as reference genes. The mean Cq values obtained from independent biological replicates are reported in Table S2.

## Figures and Tables

**Table 1:** qPCR primers.

Target Gene	Primer Sequence (5′->3′)	Reference	Amplicon Size, bp	Primer Efficiency, %
*GFP*	AGATCATATGAAACGGCATGACT	[30]	124	100.5
	ACCTTCAAACTTGACTTCAGCAC			
*slbo*	GACAAGGGCACGGATGAGTA	This study	198	100.0
	CTGCATGTAGATCTGCTTGTGT			
*Trl*	TTTCCCGCCCACAAGATAGT	This study	118	97.0
	CCAGATCGTTCGCATTGACG			
*RpL32*	CTAAGCTGTCGCACAAATGG	[59]	148	99.6
	AGGAACTTCTTGAATCCGGTG			
*Rap2l*	TCTTGGAAATATTGGACACCGC	[30]	197	102.0
	TTTGTTCGCGACTAGTAGGATG			

## Supplementary Materials

Supplementary materials can be found at https://www.mdpi.com/1422-0067/21/20/7468/s1. Figure S1. Molecular characterization of chromosomes carrying *Trl^362^* and *Trl^3609^* mutations. Table S1. Primers used for PCR verification of the *Trl* mutations and additional transposon constructs identified within the *alan shepard* (*shep*) and *couch potato* (*cpo*) genes. Table S2. RT-qPCR data: mean Cq values obtained and expression values calculated from independent biological replicates.
