# Supplementary material for: GAGA Regulates Border Cell Migration in Drosophila
Source: Int J Mol Sci. 2020 Oct 10;21(20):7468. doi: 10.3390/ijms21207468 (PMC7589894; doi:10.3390/ijms21207468)
Supplement: Supplementary file 1 [file ijms-21-07468-s001.zip › Supplementary Figure 1.pdf]

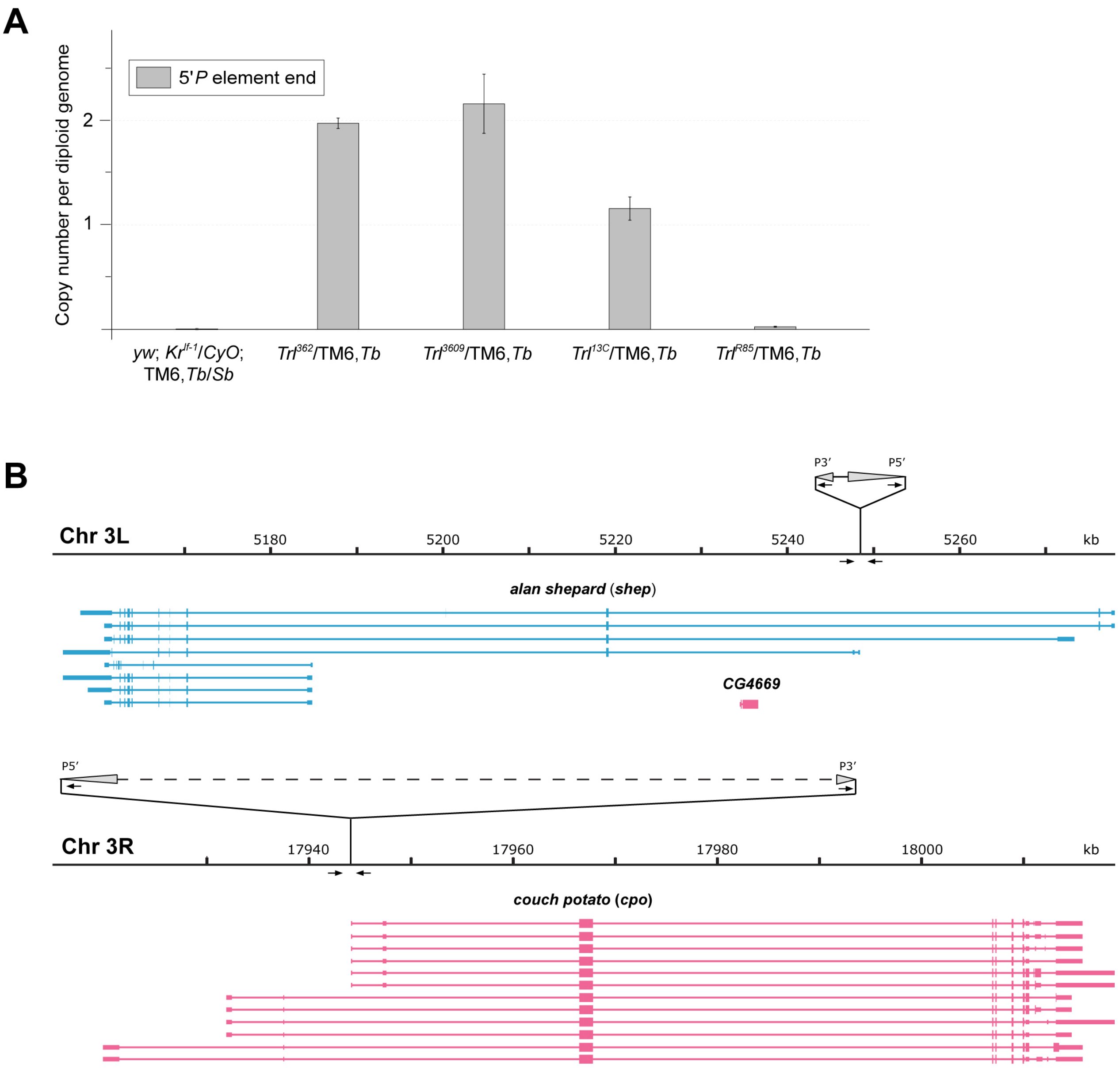

Supplementary Figure 1. Molecular characterization of chromosomes carrying *Trl*^{362} and *Trl*^{3609} mutations. (A) Copy numbers of *P* element 5′ end in the indicated fly lines as measured by qPCR. Two copies correspond to a single homozygous *P* element-based transgene in the genome. No *P* element 5′ end was found in *yw*; *Kr*$^{If-1}$/*CyO*; TM6, *Tb*/*Sb* flies used as the source of the balancer chromosomes. Error bars represent standard error of the mean. (B) Insertion sites of additional *P* element-based transgenes found in chromosomes carrying *Trl*^{362} (top) and *Trl*^{3609} (bottom) mutations. Solid horizontal black lines show segments of chromosomes flanking the insertion sites. Grey triangles represent the 5′ and 3′ *P* element ends. The transgenes are not shown to scale. *shep* and *cpo* genes affected by the insertions are located on the minus (blue) and plus (magenta) strands, respectively. Coding sequences, UTRs and introns are represented as wide bars, narrow bars and lines, respectively. Black arrows indicate the positions of primers (listed in Table S1) used for PCR verification of the transgene insertion sites.
