# Supplementary material for: GAGA Regulates Border Cell Migration in Drosophila
Source: Int J Mol Sci. 2020 Oct 10;21(20):7468. doi: 10.3390/ijms21207468 (PMC7589894; doi:10.3390/ijms21207468)
Supplement: Supplementary file 1 [file ijms-21-07468-s001.zip › Supplementary Table 1.pdf]

Supplementary Table 1. Primers used for PCR verification of the *Trl* mutations and additional transposon constructs identified within the *alan shepard* (*shep*) and *couch potato* (*cpo*) genes.

**Insertion/deletion**	***P* element end**	**Primer sequence (5′->3′)**	**Approximate amplicon size, bp**
Trl^{13C}	5′	CAATTGTTGCGCTGCCATGG<br>CACCCAAGGCTCTGCTCCCACAAT	1949
	3′	CAATCATATCGCTGTCTCACTCA<br>CGAGTGCGTGGTGTAG	1112
Trl^{362}	5′	ATGTGTTTTTCATCCGTGAAGTCG<br>CACCCAAGGCTCTGCTCCCACAAT	596
	3′	CAATCATATCGCTGTCTCACTCA<br>CAGACGTTAGTTATTAGCTC	1488
Trl^{3609}	5′	CACCCAAGGCTCTGCTCCCACAAT<br>CAGACGTTAGTTATTAGCTC	567
	3′	ATGTGTTTTTCATCCGTGAAGTCG<br>CAATCATATCGCTGTCTCACTCA	789
Trl^{R85}	-	ATTACCAAACGGTCGCATGG<br>CGAGTGCGTGGTGTAG	1643
Transposon in the *shep* gene	5′	CACCCAAGGCTCTGCTCCCACAAT<br>ACCAACCATCAAGTAACTTTGATGAAC	469
	3′	TTGTTTTGGTCGAAAAGGGATGTG<br>CAATCATATCGCTGTCTCACTCA	546
Transposon in the *cpo* gene	5′	GATAGTGTAAATTATGGCGAAATGG<br>CACCCAAGGCTCTGCTCCCACAAT	808
	3′	CAATCATATCGCTGTCTCACTCA<br>GAATAGTTTCTGGTTGCGAGATAG	479
