# Supplementary material for: GAGA Regulates Border Cell Migration in Drosophila
Source: Int J Mol Sci. 2020 Oct 10;21(20):7468. doi: 10.3390/ijms21207468 (PMC7589894; doi:10.3390/ijms21207468)
Supplement: Supplementary file 1 [file ijms-21-07468-s001.zip › Supplementary Table 2.pdf]

Supplementary Table 2. RT-qPCR data: mean Cq values obtained and expression values calculated from independent biological replicates. For each biological replicate, three technical replicates were performed.

Gene	RNA sample	Mean Cq ± SEM	Expression ± SEM	Mean expression ± SEM
slbo	*Oregon-R*	21.4033 ± 0.0100	1.0000 ± 0.0089	0.9073 ± 0.0386
		21.2402 ± 0.0281	0.9837 ± 0.0209	
		21.8146 ± 0.0262	0.7486 ± 0.0160	
		21.3075 ± 0.0193	0.8603 ± 0.0137	
		21.1520 ± 0.0265	0.9591 ± 0.0191	
		21.0597 ± 0.0168	0.8923 ± 0.0132	
	slbo^{1}/*slbo*^{1}	20.9510 ± 0.1500	0.8319 ± 0.0868	0.7892 ± 0.0393
		21.5323 ± 0.0148	0.7106 ± 0.0148	
		21.6078 ± 0.0361	0.8250 ± 0.0251	
	slbo^{1}/*slbo*^{e7b}	23.2401 ± 0.0165	0.3696 ± 0.0049	0.4078 ± 0.0273
		21.5889 ± 0.1208	0.3932 ± 0.0346	
		22.7041 ± 0.0205	0.4606 ± 0.0207	
	Trl^{R85}/*Trl*^{362}	23.4109 ± 0.0562	0.1727 ± 0.0073	0.1615 ± 0.0206
		24.0617 ± 0.0304	0.1215 ± 0.0028	
		23.4109 ± 0.0184	0.1902 ± 0.0096	
	hsp83:GAGA-519/+	21.2827 ± 0.0111	0.8448 ± 0.0090	0.9387 ± 0.0717
		21.7995 ± 0.0107	1.0795 ± 0.0115	
		21.2256 ± 0.0232	0.8918 ± 0.0154	
	slbo^{1}, *hsp83*:GAGA-519 homo	22.3169 ± 0.0312	0.7485 ± 0.0192	0.8341 ± 0.0582
		21.1566 ± 0.0031	0.8088 ± 0.0118	
		21.3691 ± 0.0158	0.9452 ± 0.0353	
	slbo^{1}, *hsp83*:GAGA-519/*slbo*^{e7b}	22.5245 ± 0.0636	0.4322 ± 0.0195	0.4251 ± 0.0191
		21.8343 ± 0.0337	0.4541 ± 0.0151	
		22.3078 ± 0.0218	0.3890 ± 0.0072	
	hsp83:GAGA-519/+; *Trl*^{R85}/*Trl*^{362}	21.7551 ± 0.1013	0.7368 ± 0.0521	0.7190 ± 0.0236
		21.5646 ± 0.0599	0.6723 ± 0.0285	
		21.5249 ± 0.0218	0.7479 ± 0.0139	
	UAS-*slbo*/+	20.7401 ± 0.0352	1.0985 ± 0.0277	0.9987 ± 0.0020
		21.1659 ± 0.0168	0.9951 ± 0.0208	
		20.9844 ± 0.0361	0.9026 ± 0.0232	
	slbo-Gal4$^{(Novo16)}$>UAS-*GFP*/UAS-*slbo*; +/+	18.6033 ± 0.0110	8.6580 ± 0.2324	9.5928 ± 0.4689
		17.7686 ± 0.0095	9.9966 ± 0.1469	
		17.4092 ± 0.0294	10.1240 ± 0.2193	
	slbo-Gal4$^{(Novo16)}$>UAS-*GFP*/UAS-*slbo*; *Trl*^{R85}/*Trl*^{362}	20.3940 ± 0.0211	2.0006 ± 0.0402	1.7862 ± 0.1685
		19.6987 ± 0.0387	1.9042 ± 0.0533	
		20.4299 ± 0.0128	1.4538 ± 0.0217	

Continued on the next page

Gene	RNA sample	Mean Cq ± SEM	Expression ± SEM	Mean expression ± SEM
Trl	*Oregon-R*	17.4477 ± 0.0131*<br>17.2076 ± 0.0261*<br>17.8512 ± 0.1051*<br>17.0084 ± 0.0308<br>17.6403 ± 0.0676<br>17.2768 ± 0.0374	1.0000 ± 0.0106*<br>1.0375 ± 0.0207*<br>0.7526 ± 0.0555*<br>1.0915 ± 0.0252<br>0.7051 ± 0.0335<br>0.7916 ± 0.0217	0.8964 ± 0.0676
	slbo^{1}/*slbo*^{1}	16.6745 ± 0.0287<br>17.3330 ± 0.0283<br>17.8322 ± 0.0369	1.0391 ± 0.0225<br>0.8414 ± 0.0225<br>0.7282 ± 0.0226	0.8696 ± 0.0908
	slbo^{1}/*slbo*^{e7b}	18.4463 ± 0.0194<br>16.3036 ± 0.0318<br>18.1831 ± 0.0043	0.6608 ± 0.0099<br>0.9884 ± 0.0345<br>0.6816 ± 0.0292	0.7769 ± 0.1059
	Trl^{R85}/*Trl*^{362}	22.3587 ± 0.0346<br>22.7990 ± 0.0051<br>22.0243 ± 0.0187	0.0231 ± 0.0007<br>0.0188 ± 0.0002<br>0.0321 ± 0.0016	0.0246 ± 0.0039
	hsp83:GAGA-519/+	16.1922 ± 0.0118<br>16.6998 ± 0.0163<br>16.5853 ± 0.0206	1.8551 ± 0.0205<br>2.3857 ± 0.0325<br>1.4335 ± 0.0223	1.8914 ± 0.2755
	slbo^{1}, *hsp83*:GAGA-519 homo	16.7044 ± 0.0363<br>15.1378 ± 0.0193<br>15.4852 ± 0.0139	2.3601 ± 0.0677<br>3.3801 ± 0.0664<br>3.5970 ± 0.1331	3.1124 ± 0.3813
	slbo^{1}, *hsp83*:GAGA-519/*slbo*^{e7b}	16.0997 ± 0.0044<br>14.8628 ± 0.0363<br>16.1232 ± 0.0227	2.3933 ± 0.0245<br>3.6730 ± 0.1271<br>1.8238 ± 0.0348	2.6300 ± 0.5468
	hsp83:GAGA-519/+; *Trl*^{R85}/*Trl*^{362}	17.1465 ± 0.0160<br>16.4828 ± 0.0324<br>17.1381 ± 0.0729	1.1584 ± 0.0159<br>1.4674 ± 0.0355<br>1.0084 ± 0.0521	1.2114 ± 0.1351
	UAS-*slbo*/+	17.2734 ± 0.0158<br>16.9452 ± 0.0245<br>16.9969 ± 0.0097	0.7827 ± 0.0100<br>1.1957 ± 0.0290<br>0.9227 ± 0.0082	0.9670 ± 0.0067
	slbo-Gal4$^{(Novo16)}$>UAS-*GFP*/UAS-*slbo*; +/+	17.7596 ± 0.0609<br>16.7658 ± 0.0044<br>16.1546 ± 0.0250	1.0014 ± 0.0495<br>1.2910 ± 0.0174<br>1.5568 ± 0.0293	1.2831 ± 0.1604
	slbo-Gal4$^{(Novo16)}$>UAS-*GFP*/UAS-*slbo*; *Trl*^{R85}/*Trl*^{362}	22.3545 ± 0.0377<br>21.4186 ± 0.0060<br>22.4472 ± 0.0779	0.0331 ± 0.0010<br>0.0373 ± 0.0003<br>0.0231 ± 0.0013	0.0312 ± 0.0042

* Only these values were used to generate barplot graph shown in Figure 3D.

Continued on the next page

Gene	RNA sample	Mean Cq ± SEM	Expression ± SEM	Mean expression ± SEM
Trl	*slbo*-Gal4$^{(chic6458)}$>UAS-*GFP*	17.7936 ± 0.0371<br>16.9880 ± 0.0468<br>17.7010 ± 0.0131	1.0000 ± 0.0372<br>0.9365 ± 0.0332<br>1.0020 ± 0.0107	0.9795 ± 0.0215
	slbo-Gal4$^{(chic6458)}$>UAS-*GFP*; *Trl*^{R85}/*Trl*^{362}	23.4067 ± 0.0218<br>22.1428 ± 0.1036<br>23.5512 ± 0.0211	0.0120 ± 0.0003<br>0.0216 ± 0.0019<br>0.0097 ± 0.0003	0.0144 ± 0.0036
	UAS-*GFP*/+	17.2601 ± 0.0144<br>17.0236 ± 0.0207<br>17.5410 ± 0.1272	0.7044 ± 0.0101<br>0.7739 ± 0.0166<br>0.5575 ± 0.0496	0.6786 ± 0.0638
	slbo-Gal4$^{(Novo11)}$>UAS-*GFP*/+; *Trl*^{R85}/*Trl*^{362}	22.4464 ± 0.0288<br>22.2090 ± 0.0278<br>22.5509 ± 0.0379	0.0270 ± 0.0006<br>0.0214 ± 0.0004<br>0.0240 ± 0.0006	0.0241 ± 0.0016
	slbo-Gal4$^{(Novo16)}$>UAS-*GFP*/+; *Trl*^{R85}/*Trl*^{362}	21.5352 ± 0.0262<br>22.9905 ± 0.0117<br>21.4024 ± 0.0575	0.0353 ± 0.0008<br>0.0297 ± 0.0004<br>0.0268 ± 0.0016	0.0306 ± 0.0025
	slbo-Gal4$^{(Novo22)}$>UAS-*GFP*/+; *Trl*^{R85}/*Trl*^{362}	23.2754 ± 0.0385<br>22.1463 ± 0.0442<br>23.0400 ± 0.0302	0.0216 ± 0.0006<br>0.0225 ± 0.0007<br>0.0132 ± 0.0003	0.0191 ± 0.0029
	slbo-Gal4$^{(Novo11)}$>UAS-*GFP*/+; +/+	17.5681 ± 0.0118<br>16.1867 ± 0.0322<br>18.6205 ± 0.0240	0.5668 ± 0.0053<br>1.0332 ± 0.0258<br>0.5736 ± 0.0096	0.7245 ± 0.1543
	slbo-Gal4$^{(Novo16)}$>UAS-*GFP*/+; +/+	17.1742 ± 0.0150<br>17.0610 ± 0.0387<br>17.1287 ± 0.0222	0.7765 ± 0.0088<br>0.9510 ± 0.0295<br>1.2057 ± 0.0192	0.9777 ± 0.1246
	slbo-Gal4$^{(Novo22)}$>UAS-*GFP*/+; +/+	17.5922 ± 0.0237<br>16.8562 ± 0.0017<br>17.5055 ± 0.0190	0.6747 ± 0.0114<br>0.8599 ± 0.0066<br>0.5862 ± 0.0081	0.7069 ± 0.0807

Continued on the next page

Gene	RNA sample	Mean Cq ± SEM*	Expression ± SEM	Mean expression ± SEM
GFP	*slbo*-Gal4$^{(chic6458)}$>UAS-*GFP*	20.0186 ± 0.0315<br>19.2386 ± 0.1306<br>19.8383 ± 0.0058	1.0000 ± 0.0276<br>0.9201 ± 0.0844<br>1.0648 ± 0.0074	0.9949 ± 0.0419
	slbo-Gal4$^{(chic6458)}$>UAS-*GFP*; *Trl*^{R85}/*Trl*^{362}	20.1867 ± 0.0164<br>19.7377 ± 0.1247<br>20.0563 ± 0.0337	0.4183 ± 0.0096<br>0.5348 ± 0.0527<br>0.5095 ± 0.0168	0.4875 ± 0.0354
	UAS-*GFP*/+	25.5306 ± 0.0299<br>25.6610 ± 0.1191<br>26.2022 ± 0.0424	0.0107 ± 0.0002<br>0.0091 ± 0.0008<br>0.0064 ± 0.0002	0.0087 ± 0.0012
	slbo-Gal4$^{(Novo11)}$>UAS-*GFP*/+; *Trl*^{R85}/*Trl*^{362}	19.1414 ± 0.1227<br>18.4283 ± 0.1266<br>19.3489 ± 0.0039	1.2497 ± 0.1074<br>1.3720 ± 0.1208<br>1.0335 ± 0.0063	1.2184 ± 0.0990
	slbo-Gal4$^{(Novo16)}$>UAS-*GFP*/+; *Trl*^{R85}/*Trl*^{362}	19.6704 ± 0.0279<br>20.9456 ± 0.0167<br>19.0771 ± 0.0202	0.6013 ± 0.0143<br>0.5720 ± 0.0093<br>0.6272 ± 0.0285	0.6001 ± 0.0159
	slbo-Gal4$^{(Novo22)}$>UAS-*GFP*/+; *Trl*^{R85}/*Trl*^{362}	19.7699 ± 0.0132<br>18.9388 ± 0.0397<br>20.1770 ± 0.0107	1.1476 ± 0.0167<br>0.9709 ± 0.0271<br>0.4504 ± 0.0045	0.8563 ± 0.2092
	slbo-Gal4$^{(Novo11)}$>UAS-*GFP*/+; +/+	17.8585 ± 0.0184<br>17.0155 ± 0.0044<br>19.3416 ± 0.0223	2.1668 ± 0.0294<br>2.7195 ± 0.0316<br>1.6269 ± 0.0253	2.1711 ± 0.3154
	slbo-Gal4$^{(Novo16)}$>UAS-*GFP*/+; +/+	17.5969 ± 0.0247<br>17.2318 ± 0.0212<br>18.1911 ± 0.0075	2.7082 ± 0.0480<br>3.9495 ± 0.0844<br>2.6990 ± 0.0177	3.1189 ± 0.4153
	slbo-Gal4$^{(Novo22)}$>UAS-*GFP*/+; +/+	18.5000 ± 0.0219<br>17.2628 ± 0.0215<br>17.7933 ± 0.0228	1.6812 ± 0.0264<br>3.0328 ± 0.0506<br>2.2449 ± 0.0367	2.3196 ± 0.3920
	Oregon-R	34.0374 ± 0.0217<br>34.0883 ± 0.0211<br>35.1408 ± 0.0220	0.0000 ± 0.0000<br>0.0001 ± 0.0000<br>0.0001 ± 0.0000	0.0001 ± 0.0000

Continued on the next page

Gene	RNA sample	Mean Cq ± SEM
RpL32	*Oregon-R*	11.3093 ± 0.0243*<br>11.1168 ± 0.0239*<br>11.2807 ± 0.0278*<br>11.0365 ± 0.0199<br>10.9731 ± 0.0161<br>10.4530 ± 0.0240
	slbo^{1}/*slbo*^{1}	10.4235 ± 0.0231<br>11.0258 ± 0.0157<br>11.1129 ± 0.0136
	slbo^{1}/*slbo*^{e7b}	11.3562 ± 0.0159<br>11.2119 ± 0.0264<br>10.0382 ± 0.0767
	Trl^{R85}/*Trl*^{362}	10.6344 ± 0.0405<br>11.0197 ± 0.0279<br>10.7844 ± 0.1400
	hsp83:GAGA-519/+	10.9918 ± 0.0084<br>11.8778 ± 0.0216<br>11.0085 ± 0.0159
	slbo^{1}, *hsp83*:GAGA-519 homo	11.5087 ± 0.0369<br>10.4690 ± 0.0345<br>11.0463 ± 0.1026
	slbo^{1}, *hsp83*:GAGA-519/*slbo*^{e7b}	11.0739 ± 0.0045<br>10.5279 ± 0.0603<br>10.5635 ± 0.0308
	hsp83:GAGA-519/+; *Trl*^{R85}/*Trl*^{362}	11.0243 ± 0.0188<br>10.6500 ± 0.0260<br>10.7027 ± 0.0259
	UAS-*slbo*/+	10.4712 ± 0.0170<br>11.0460 ± 0.0493<br>10.6375 ± 0.0060
	slbo-Gal4$^{(Novo16)}$>UAS-*GFP*/UAS-*slbo*; +/+	11.6797 ± 0.0695<br>11.0932 ± 0.0371<br>10.7723 ± 0.0090
	slbo-Gal4$^{(Novo16)}$>UAS-*GFP*/UAS-*slbo*; *Trl*^{R85}/*Trl*^{362}	11.3647 ± 0.0298<br>10.5661 ± 0.0148<br>10.6456 ± 0.0337

* Only these values were used to generate barplot graph shown in Figure 3D.

Continued on the next page

Gene	RNA sample	Mean Cq ± SEM
RpL32	*slbo*-Gal4$^{(chic6458)}$>UAS-*GFP*	12.2724 ± 0.0186<br>11.2995 ± 0.0409<br>12.0544 ± 0.0135
	slbo-Gal4$^{(chic6458)}$>UAS-*GFP*; Trl^{R85}/Trl^{362}	11.2079 ± 0.0381<br>10.9230 ± 0.1358<br>11.2168 ± 0.0650
	UAS-*GFP*/+	11.1445 ± 0.0117<br>11.0446 ± 0.0137<br>11.1408 ± 0.0341
	slbo-Gal4$^{(Novo11)}$>UAS-*GFP*/+; Trl^{R85}/Trl^{362}	11.8304 ± 0.0328<br>11.1455 ± 0.0169<br>11.7485 ± 0.0059
	slbo-Gal4$^{(Novo16)}$>UAS-*GFP*/+; Trl^{R85}/Trl^{362}	11.1844 ± 0.0344<br>12.2663 ± 0.0294<br>10.4702 ± 0.0256
	slbo-Gal4$^{(Novo22)}$>UAS-*GFP*/+; Trl^{R85}/Trl^{362}	11.2351 ± 0.0144<br>12.2131 ± 0.0276<br>11.1389 ± 0.0118
	slbo-Gal4$^{(Novo11)}$>UAS-*GFP*/+; +/+	11.0914 ± 0.0003<br>10.4621 ± 0.0257<br>12.1531 ± 0.0026
	slbo-Gal4$^{(Novo16)}$>UAS-*GFP*/+; +/+	11.2254 ± 0.0102<br>11.2349 ± 0.0438<br>11.8066 ± 0.0042
	slbo-Gal4$^{(Novo22)}$>UAS-*GFP*/+; +/+	11.4216 ± 0.0056<br>11.0740 ± 0.0217<br>11.1010 ± 0.0092

Continued on the next page

Gene	RNA sample	Mean Cq ± SEM
Rap2l	*Oregon-R*	15.2012 ± 0.0069*<br>15.0200 ± 0.0053*<br>15.2168 ± 0.0166*<br>14.8481 ± 0.0151<br>14.9144 ± 0.0150<br>15.0417 ± 0.0108
	slbo^{1}/*slbo*^{1}	14.6515 ± 0.0087<br>14.7571 ± 0.0497<br>15.2516 ± 0.0485
	slbo^{1}/*slbo*^{e7b}	15.8760 ± 0.0103<br>14.1505 ± 0.0152<br>15.5834 ± 0.1203
	Trl^{R85}/*Trl*^{362}	14.8235 ± 0.0227<br>14.7265 ± 0.0086<br>14.9525 ± 0.0071
	hsp83:GAGA-519/+	14.7908 ± 0.0197<br>15.6458 ± 0.0040<br>14.8163 ± 0.0084
	slbo^{1}, *hsp83*:GAGA-519 homo	15.9930 ± 0.0139<br>14.9359 ± 0.0232<br>15.2330 ± 0.0098
	slbo^{1}, *hsp83*:GAGA-519/*slbo*^{e7b}	15.1787 ± 0.0279<br>14.5668 ± 0.0325<br>15.0320 ± 0.0044
	hsp83:GAGA-519/+; *Trl*^{R85}/*Trl*^{362}	15.2283 ± 0.0140<br>14.9846 ± 0.0005<br>15.1858 ± 0.0169
	UAS-*slbo*/+	14.9039 ± 0.0085<br>14.9754 ± 0.0086<br>14.7395 ± 0.0158
	slbo-Gal4$^{(Novo16)}$>UAS-*GFP*/UAS-*slbo*; +/+	15.4589 ± 0.0262<br>14.7908 ± 0.0076<br>14.4294 ± 0.0193
	slbo-Gal4$^{(Novo16)}$>UAS-*GFP*/UAS-*slbo*; *Trl*^{R85}/*Trl*^{362}	15.1281 ± 0.0264<br>14.3935 ± 0.0172<br>14.9977 ± 0.0075

* Only these values were used to generate barplot graph shown in Figure 3D.

Continued on the next page

Gene	RNA sample	Mean Cq ± SEM
Rap2l	*slbo*-Gal4$^{(chic6458)}$>UAS-*GFP*	16.1519 ± 0.0448<br>15.3243 ± 0.0073<br>16.1905 ± 0.0092
	slbo-Gal4$^{(chic6458)}$>UAS-*GFP*; *Trl*^{R85}/*Trl*^{362}	15.2957 ± 0.0434<br>15.1335 ± 0.0117<br>15.3372 ± 0.0150
	UAS-*GFP*/+	15.2017 ± 0.0276<br>15.1000 ± 0.0439<br>15.0925 ± 0.0101
	slbo-Gal4$^{(Novo11)}$>UAS-*GFP*/+; *Trl*^{R85}/*Trl*^{362}	15.4826 ± 0.0149<br>15.0108 ± 0.0136<br>15.4315 ± 0.0147
	slbo-Gal4$^{(Novo16)}$>UAS-*GFP*/+; *Trl*^{R85}/*Trl*^{362}	15.0756 ± 0.0195<br>16.4002 ± 0.0143<br>14.7250 ± 0.1219
	slbo-Gal4$^{(Novo22)}$>UAS-*GFP*/+; *Trl*^{R85}/*Trl*^{362}	15.2049 ± 0.0134<br>16.1109 ± 0.0170<br>15.0406 ± 0.0056
	slbo-Gal4$^{(Novo11)}$>UAS-*GFP*/+; +/+	15.2438 ± 0.0134<br>14.8426 ± 0.0196<br>16.3214 ± 0.0023
	slbo-Gal4$^{(Novo16)}$>UAS-*GFP*/+; +/+	15.2302 ± 0.0080<br>15.5792 ± 0.0096<br>15.8276 ± 0.0108
	slbo-Gal4$^{(Novo22)}$>UAS-*GFP*/+; +/+	15.4644 ± 0.0097<br>15.0399 ± 0.0018<br>15.2059 ± 0.0079
